# High prevalence of gastric intestinal metaplasia detected by confocal laser endomicroscopy in Zambian adults

**DOI:** 10.1371/journal.pone.0184272

**Published:** 2017-09-08

**Authors:** Violet Kayamba, Aaron Shibemba, Kanekwa Zyambo, Douglas C. Heimburger, Douglas R. Morgan, Paul Kelly

**Affiliations:** 1 Tropical Gastroenterology & Nutrition group, Department of Internal Medicine, University of Zambia School of Medicine, Lusaka, Zambia; 2 Cancer Diseases Hospital, Pathology section, Lusaka, Zambia; 3 Vanderbilt Institute for Global Health, Vanderbilt University Medical Center, Nashville, Tennessee, United States of America; 4 Blizard Institute, Barts & The London School of Medicine and Dentistry, Queen Mary University of London, London, United Kingdom; Central University of Tamil Nadu, INDIA

## Abstract

**Background:**

Confocal laser endomicroscopy (CLE) may increase the detection of gastric premalignant lesions, and facilitate targeted biopsies for histology. The study aim was to analyse premalignant lesions in Zambian adults using CLE.

**Methods:**

Using CLE and histology we analysed the antral mucosa for gastric premalignant lesions in asymptomatic adults living with HIV and in HIV seronegative adults. Fasting gastric pH and the presence of *Helicobacter pylori (H*. *pylori)* were also evaluated.

**Results:**

We enrolled 84 HIV seropositive participants (median age 43 years; 55 (65%) female), of whom 32 (38%) were anti-retroviral therapy (ART)-naïve. Also enrolled were 22 HIV seronegative controls (median age 39 years, 12 (55%) females). Hypochlorhydria was found in 48 (57%) HIV positive and 8 (38%) HIV negative controls (*P* = 0.14). Detection of gastric intestinal metaplasia (GIM) was higher (*P* = 0.007) using CLE (49, 54%) than histology (9, 9%) and, using CLE, GIM was similar between HIV positive (41, 60%) and negative groups (8, 36%; *P* = 0.08). Gastric luminal fluorescein leakage was significantly associated with the presence of GIM [OR 8.2; 95% CI 2.5–31, *P*<0.001].

**Conclusion:**

CLE is useful for the detection of GIM, and luminal fluorescein leakage may represent a novel CLE marker for GIM. GIM is common in Zambian adults, and is highly prevalent irrespective of HIV infection or use of ART.

## Background

Confocal laser endomicroscopy (CLE) may have higher sensitivity and specificity for detecting gastric intestinal metaplasia (GIM) than conventional white light endoscopy (WLE) [1]. The technique allows real-time high resolution and magnification of the mucosa of up to 1000 times, “virtual histology” [2]. There are two types of CLE: endoscope-based eCLE and probe-based pCLE. Both systems require contrast and use laser light for excitation. eCLE can capture images at varying depth and it has a wider field of view and higher resolution, while pCLE has the advantage of being applicable through any type of endoscope [3,4]. The detection of GIM using WLE guided random biopsies is challenging as consistent characteristic features are lacking, and GIM tends to be patchy [5,6]. CLE may allow thorough mapping of GIM to facilitate targeting of biopsies.

Sub-Saharan Africa (SAA) accounts for over two-thirds of the Human Immunodeficiency virus (HIV) global burden [7]. With widely available antiretroviral therapy (ART), persons living with HIV (PLHIV) are living longer and healthier lives, and non-communicable diseases such as gastric cancer are now emerging as significant contributors to morbidity and mortality in the region. In Zambia, there has been a steady increase in the diagnosis of gastric cancer among young adults [8,9], the same age group that is affected by HIV infection. The influence of HIV infection on the gastrointestinal mucosa has been a subject of extensive investigation, but is still not fully understood. Gut-associated lymphoid tissue continues to harbour HIV in persons who have been well suppressed on ART [10], with the gastrointestinal tract being the major site for HIV replication [11]. The single most important risk factor for gastric cancer is *Helicobacter pylori (H*. *pylori)* infection and it has been reported to be lower in HIV infected individuals [12,13,14,15], but the risk of cancer is not reduced and may be increased. In addition, *H*. *pylori*-induced gastric lesions are reported to be more severe in HIV infection [16]. The prevalence of premalignant lesions such as GIM, atrophy, or dysplasia in PLHIV is currently unknown but will be important for the long-term health of these individuals.

Hypochlorhydria (gastric pH greater than 4) is more common in PLHIV but the mechanism is not clear [9,17]. Evidence of glandular atrophy causing hypochlorhydria in these patients is lacking [9,18], suggesting that it could be occurring by another mechanism such as immune modulation, for example by interleukin-1β. The occurrence of hypochlorhydria in HIV infection is not influenced by the clinical stage of the disease [19] or the CD4 count [20]. It is possible that the lower prevalence of *H*. *pylori* in PLHIV results from persistent hypochlorhydria.

Our study aim was to explore the utility of CLE for the detection of gastric premalignant lesions in PLHIV with an HIV negative control group. We mapped the gastric antrum into specified areas and compared with histology. The University of Zambia Biomedical Research Ethics committee (Ref no. 004-04-14) and the National Health Research Authority approved this study.

## Methods

### Participant enrolment

The study was conducted at the University Teaching Hospital (UTH) in Lusaka, Zambia. It was a cross-sectional study, with HIV positive participants recruited from the UTH HIV outpatient clinic. Asymptomatic HIV infected adults (age ≥ 18 years) were enrolled in two groups: ART-naïve and on ART with full viral suppression (“ART-treated”). After a detailed explanation, all participants gave informed written consent. Exclusion criteria included ART failure, a history of significant gastrointestinal disease (e.g., active peptic ulcer, gastric surgery) and significant co-morbidities (e.g., chronic liver or kidney disease). To compare the CLE findings HIV negative dyspeptic patients were enrolled from the endoscopy unit.

### Upper gastrointestinal endoscopy

We employed both white light endoscopy (WLE) using a Pentax EG-2990i instrument, and endoscope-based confocal laser endomicroscopy (CLE) using a Pentax EC 3870CLK instrument. A full assessment of the upper gastrointestinal tract was carried out first using WLE according to a standard protocol. Gastric fluid was aspirated for pH determination, using pH paper test strips (Sigma Chemical Company St Louis, USA). Hypochlorhydria was defined as gastric pH>4. CLE was used to image the antrum after administering 5–10 ml of 2% fluorescein sodium intravenously [17]. The antrum was carefully mapped into eleven distinct areas for CLE and biopsy: Areas 1, 2 and 3 were on the incisura, areas 4, 5 and 6 were between the incisura and the pyloric opening, and areas 7, 8 and 9 were on the greater curve of the antrum. Area 10 was near the antral-corporal junction on the greater curve, and area 11 was proximal to the incisura. S1 Table shows how our mapping compares with the systemic alphanumeric-coded endoscopy (SACE) [21]. Between 10 and 30 CLE images were obtained from each site, an average of 200 per patient, and all images free of motion artefact were saved. Non-targeted biopsies were obtained from areas 1, 2, 3 and 5 in separate formalin containers.

### Assessment of images obtained during confocal laser endomicroscopy

The CLE images were analysed by a study endoscopist (V.K.) who was blinded to the pathology results, searching for GIM and luminal fluorescein leakage. CLE criteria for diagnosing GIM, derived from the existing literature [22], included presence of goblet cells, columnar absorptive cells with brush border, or villus-like appearance with the interstitium in the centre [22,23]. We also compared gastric images to duodenal images obtained in a previous study [17]. Individuals with GIM in any one of the eleven areas described above were considered to be positive for GIM. To check for intra-observer variability, the images were re-analysed (by V.K.) after four weeks. To estimate inter-observer agreement, a second blinded endoscopist (P.K.) analysed images from area 6, where GIM was detected most frequently.

### Histopathology

Gastric biopsies were stained with haematoxylin and eosin, with Giemsa special staining for *H*. *pylori*. An experienced histopathologist (A.S.) characterized gastric histology by considering infiltration of the lamina propria by acute or chronic inflammatory cells to diagnose active or chronic non-atrophic gastritis (NAG). The presence of gastritis with increased distance between the individual glands (glandular loss) and the condensation of reticulin fibres in the lamina propria was used to diagnose chronic atrophic gastritis (CAG) [22]. The presence of goblet cells, absorptive (brush border) cells and/or Paneth cells was used to define GIM [24].

### Laboratory analysis

Serological analysis for *H*. *pylori* was carried out using the Biohit GastroPanel^®^ ELISA kits, (Biohit Helsinki, Finland). Per the manufacturer’s protocol samples with enzyme immunounits 30 or greater were considered positive for the presence of *H*. *pylori* IgG antibodies. To test for the presence of HIV infection, the Uni-Gold^™^ kit was used (Trinity Biotech, Wicklow, Ireland). The HIV viral load in plasma was measured with the COBAS AmpliPrep/COBAS TaqMan HIV-1 Test, version 2 (Roche Molecular Systems, Branchburg, USA).

### Statistical analysis

Categorical variables were summarized using proportions; medians and interquartile ranges were calculated for continuous variables. Binary variables were compared using Fisher’s exact test and presented as odds ratios with 95% confidence intervals. The Kruskal-Wallis test was used to compare continuous variables. Correlations were analysed using Spearman rank’s correlation. We calculated the Kappa-statistic to measure inter-rater agreement. Both univariable and multivariable analyses were used to assess for various associations. In all instances, a two-sided *P* value of <0.05 was considered statistically significant. Statistical analysis was done in STATA 13 (College Station, TX, USA).

## Results

**Fig 1:**
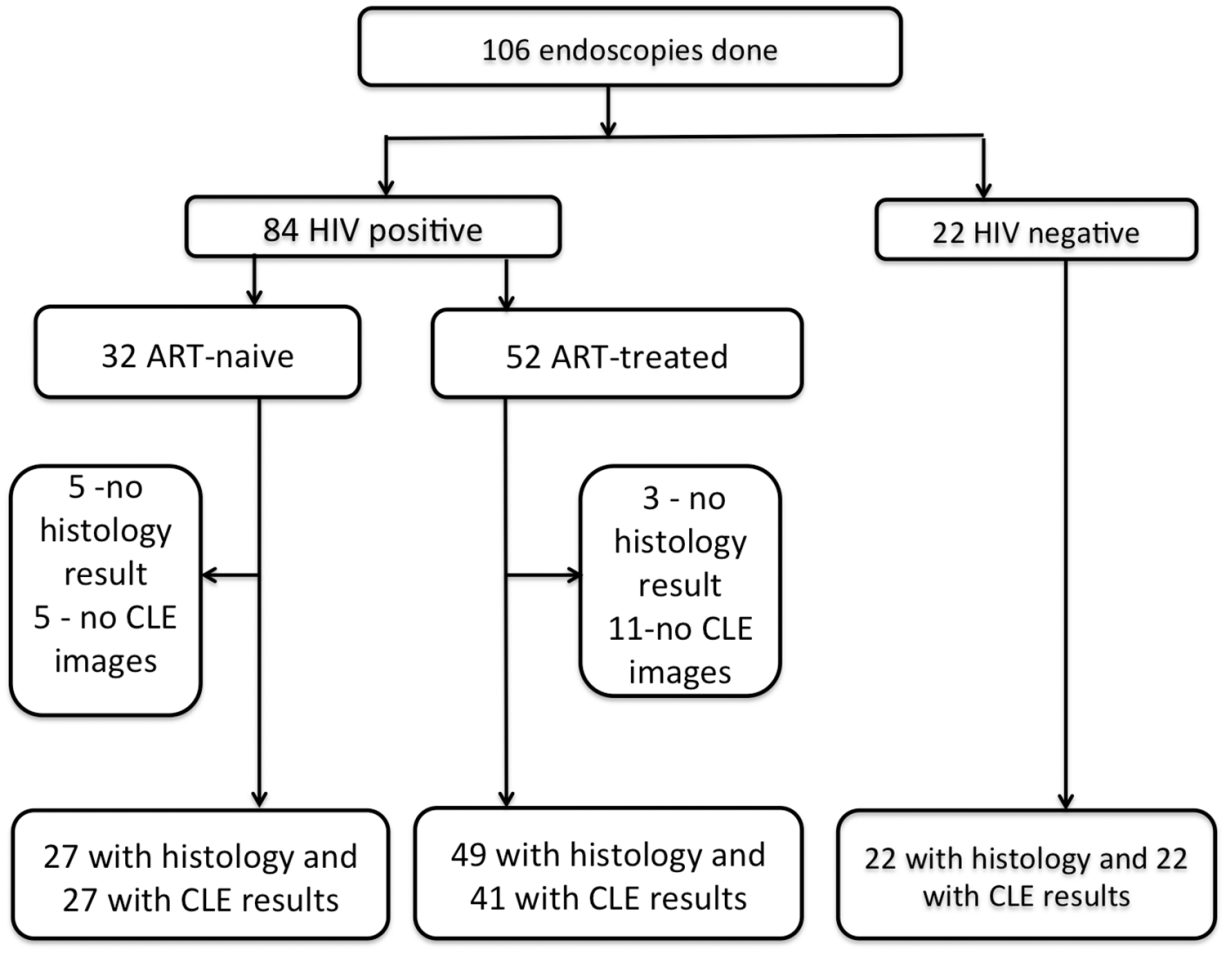
Flow chart showing the availability of data for analysis.

We enrolled and conducted endoscopy on 106 individuals, including 84 asymptomatic HIV positive and 22 HIV negative controls (Fig 1). All who agreed to participate in the study met the inclusion criteria and none were excluded. The median age for the HIV positive group was 43 years (45 years for ART-treated and 36 years for the ART-naïve) and 39 for the HIV negative group. The ART-treated group was significantly older but further analysis showed that age had no influence on the presence of either atrophy or intestinal metaplasia. As expected, those on ART had significantly higher median CD4 counts than those who were ART-naive: 522 compared to 297 cells/μl, *P* = 0.001 (Table 1). Among the ART-naïve participants, 5/32 (16%) had abnormal findings on WLE: gastric ulcers (n = 3), oesophageal candidiasis and gastric Kaposi’s sarcoma. In the ART-treated group 8/52 participants had abnormalities on WLE: gastric ulcers (n = 2), inflamed mucosa (n = 5) and oesophageal varices (n = 1) (Table 1). Four HIV negative patients had visible endoscopic abnormalities with duodenal ulcers (n = 3) and gastric polyp (n = 1). None of the ulcers were malignant lesions.

**Table 1:** Comparison of the basic characteristics of HIV positive ART-treated, HIV positive ART-naïve and HIV negative individuals.

	ART-treated	ART-naive	HIV negative	P
**Number**	52	32	22	
**Females** **Males**	35 (67) 17 (33)	20 (62) 12 (38)	12 (55) 10 (45)	0.56
**Age (median; IQR)**	45 (40–52)	36 (30–44)	39 (32–44)	<0.001
**BMI, median (IQR)**	23 (21–27)	21.5 (20–23)	24 (23–28)	0.05
**Abnormalities on WLE**	18 (15)	5 (16)	4 (18)	0.94
**HIV viral load, copies/μL** **Median (IQR)**	0 (0.0–0.0)	47475 (3275–229540)	-	Selection criterion
**Years on ART (median; IQR)**	10 (7–11)	0 (0.0–0.0)	-	Selection criterion
**CD4 count, cells/μL median (IQR)**	522 (353–632)	297 (94–354)	-	0.001

ART, anti-retroviral therapy; WLE, white light endoscopy; IQR, inter-quartile range.

### Confocal laser endomicroscopy reveals patchy but widespread distribution of gastric intestinal metaplasia

**Fig 2:**
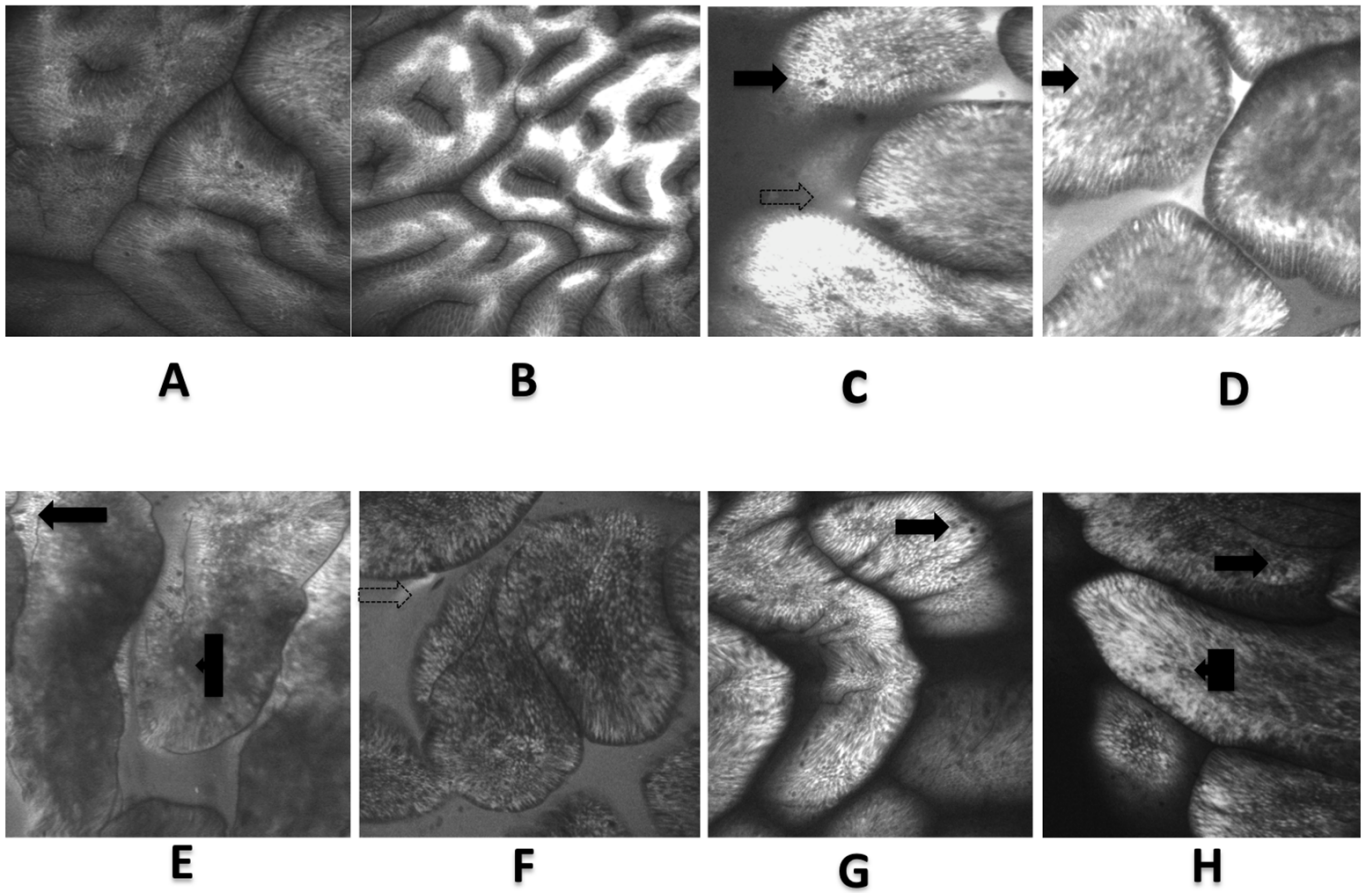
Confocal laser endomicroscopy images: A- normal antrum, B- normal antrum showing confinement of fluorescein within the mucosa. C to E shows CLE images of gastric intestinal metaplasia and F to H: comparative CLE images of the duodenum. Visible in both sets are the presence of villus-like structures, goblet cells (solid arrows), intraepithelial lymphocytes (box arrows), plumes (dotted arrows) and a visible brush borders.

We obtained an average of 200 CLE images per patient from the 11 areas of the antrum. Normal images were noted as shown in Fig 2. There was significant intra-observer agreement with an overall mean kappa statistic of 0.73 and an agreement of 94% for image analyses carried out four weeks apart (S2 Table). In addition, the inter-rater agreement between two observers (V.K. and P.K.) for area 6 was strong, with a kappa statistic of 0.86. We compared our CLE images with a large (8,200) collection of images from the small intestine [17] and found very clear similarities (Fig 2), giving considerable confidence in our endomicroscopic diagnosis of GIM. Many of the endomicroscopic features of enteropathic intestinal mucosa such as plumes and epithelial damage [22,23] were also visible in the GIM images.

Overall, 41(60%) HIV positive and 8 (36%) HIV negative individuals had GIM on CLE, a difference which was not statistically different (*P* = 0.08). Among the HIV positive group, 27 (40%) had GIM in more than one of the eleven areas, and none had it in all the areas. In area 6, GIM was diagnosed in 35% of these participants. Analysis using a non-parametric trend test showed that the number of sites showing GIM on CLE images was not affected by age, sex, ART use, gastric pH, antral atrophy, *H*. *pylori* infection, CD4 count or viral load (S3 Table).

### Histologic diagnosis of gastric intestinal metaplasia, non-atrophic gastritis chronic atrophic gastritis and *Helicobacter pylori* infection

On histological evaluation of antral biopsies of HIV positive individuals, 7 (9%) had GIM, 62 (82%) NAG and 4 (5%) CAG. In comparison, 2 (9%) of the HIV negative individuals had GIM, 20 (91%) NAG and none had CAG. There was no statistically significant difference in the occurrence of these lesions in the two groups. Combining all the data, GIM was found by histology in 9 (9%) participants compared to 49 (54%) by CLE (*P* = 0.007). All participants with GIM on histology also had it on CLE. Analysis of specific of HIV positive individuals showed that six of them had GIM in only one of the four areas sampled. One patient had GIM in three of the four biopsied areas, corresponding well with CLE findings, which showed GIM in nine out of eleven sites in that individual. Comparing histology and CLE in the four biopsied areas (1, 2, 3 and 5), a significantly higher number of participants were found to have GIM by CLE (22/68, 32%) than by histology (7/69, 9%; *P* = 0.01; S4 Table).

**Fig 3:**
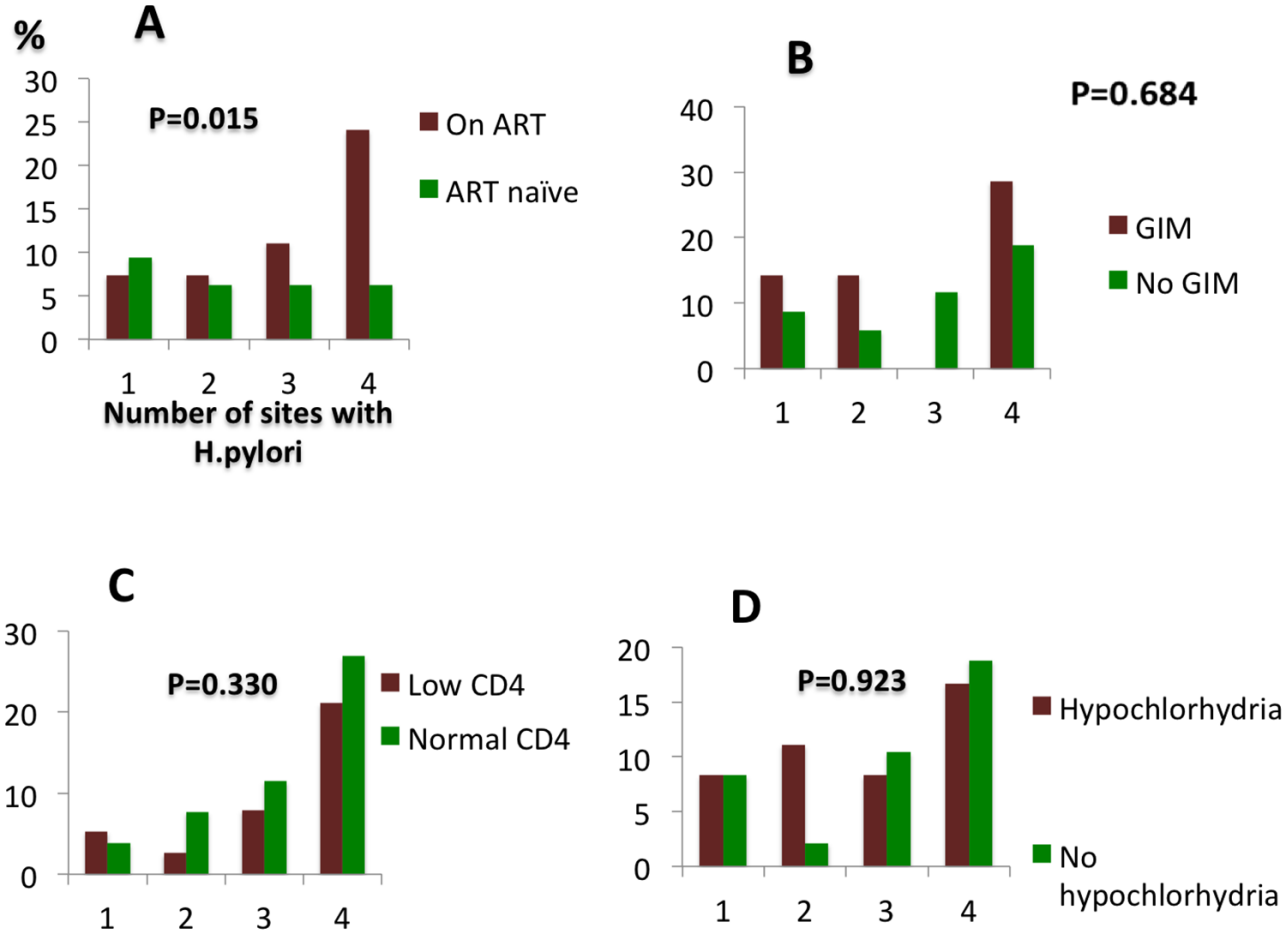
Number of antral sites in HIV positive individuals colonized by H. pylori seen in relation to the following variables: A use of ART; B gastric intestinal metaplasia (detected on histology); C Low CD4 count (less than 500 cells/ul); D Hypochlorhydria (x-axes show the number of sites with H. pylori infection and y-axes are the percentages).

Overall, 40% of all the participants had active *H*. *pylori* infection, with the ART-treated group having a significantly higher proportion (Table 2). *H*. *pylori* infection was associated with active NAG in the HIV negative group (*P* = 0.009) but not in either the ART-treated (*P* = 0.49) or the ART-naïve (*P* = 0.1). The association between active NAG and *H*. *pylori* was also not seen using serology in HIV positive individuals [OR 0.3; 95% CI 0.04–34.1, *P* = 0.54]. Comparing the two HIV positive groups on their own, the number of sites with *H*. *pylori* detected on histology was greater in patients treated with ART (Fig 3).

**Table 2:** Gastric pathology in HIV positive ART-treated, HIV positive ART- naïve and HIV negative individuals.

Global mucosal diagnosis	ART treated n (%)	ART naïve n (%)	HIV negative n (%)	P-value
**CLE completed**	**n = 41**	**n = 27**	**n = 22**	
**Histology completed**	**n = 49**	**n = 27**	**n = 22**	
**Intestinal metaplasia (Histology)**	6 (12)	1 (4)	2 (9)	0.60
**Intestinal metaplasia (CLE)**	25 (61)	16 (59)	8 (36)	0.16
**Hypochlorhydria**	29 (56)	19 (59)	7 (33)	0.15
**Fluorescein leakage**	13 (32)	10 (40)	8 (36)	0.78
***H*. *pylori* (Histology) Current infection**	26 (53)	9 (33)	5 (23)	0.04
**Chronic non-atrophic gastritis (Histology)**	34 (69)	24 (89)	15 (68)	0.13
**Active non-atrophic gastritis (Histology)**	2 (4)	2 (7)	5 (23)	0.05
**Antral atrophy (Histology)**	4 (8)	0(0)	0 (0)	0.18

### ART does not influence hypochlorhydria in HIV infection

Hypochlorhydria was present in 48 (57%) HIV positive and 7 (33%) HIV negative individuals (*P* = 0.08). However, 20 (91%) of the HIV negative and none of the HIV positive individuals gave a history of recent use of proton pump inhibitors prior to enrolment. The median gastric pH was 4.8; IQR 2–6, in the ART-naïve group and 5.5; IQR 2–6, in the ART-treated group.

### Fluorescein leakage is associated with gastric intestinal metaplasia

Luminal leakage of fluorescein was noted in 23 (34%) HIV positive and 8 (36%) HIV negative individuals (*P* = 1.0). Analysis of the pooled data showed a strong association between GIM and fluorescein leakage [OR 8.2; 95% CI 2.5–31, *P*<0.001] (Table 3). The positive predictive value was 91% and the negative predictive value was 65%. This yielded a specificity of 89% and sensitivity of 69%.

**Table 3:** The association between fluorescein leakage and gastric mucosal lesions (including both HIV positive and negative controls).

Global mucosal diagnosis		Fluorescein leakage	No fluorescein leakage	OR; 95% CI	P-value
**GIM on CLE**	Present	26	22	8.2(2.5–31)	<0.001
	Absent	5	35		
**GIM on histology**	Present	5	3	3.5(0.6–24.2)	0.12
	Absent	25	53		
NAG*	Present	24	48	0.7(0.2–2.6)	0.55
	Absent	6	8		
CAG*	Present	1	2	0.1(0.01–18.6)	1
	Absent	29	54		

*For these two groups, participants with gastric intestinal metaplasia (GIM) were removed

## Discussion

We set out to investigate the utility of CLE for detecting GIM, an important premalignant lesion, in both HIV infected and uninfected individuals. Detection of GIM was significantly higher using CLE than histology of biopsies obtained under the guidance of ordinary WLE. We also found that the occurrence of GIM was not affected by either HIV infection or the use of ART for viral suppression. In addition, fluorescein leakage by CLE was strongly associated with GIM and this may be a unique additional feature for the CLE diagnosis of GIM.

As histology is the currently accepted gold standard for the diagnosis of GIM, we considered the possibility of GIM over-diagnosis by CLE. We therefore compared the images of GIM to those of the duodenum and found that they were indistinguishable, allowing a high degree of confidence that these gastric epithelia were of the intestinal type. The features of GIM identification that we employed were as reported by other investigators [22] with low intra- and inter-observer variability. Systematically mapping the antrum provided further evidence of the patchy nature of GIM [5,6], as none of the participants had it in all eleven areas. This underscores the difficulty in identifying GIM with non-targeted biopsies. The highest yield of GIM was from near the incisura, suggesting that this would be the best area for random sampling. Some of our CLE images showed intestinal mucosal breaches similar to those seen in inflammatory bowel disease [23] and environmental enteropathy [17], confirming that the metaplastic epithelium has impaired barrier function. We therefore speculate that bacterial or endotoxin translocation could be occurring through the gastric mucosa of persons with GIM, resulting in systemic inflammatory response, but this requires further investigation. CLE has the potential of compensating for inherent limitations of conventional endoscopic and histopathological techniques [25]. However, the high capital and maintenance costs of CLE limit its utility in low resource settings. There is an urgent need to find cost-effective strategies for detecting gastric premalignant lesions in these populations. Using histology, the prevalence of GIM detected in this study was not significantly different from other African countries, such as Cameroon 6.3% [26], Morocco 15% [27] and Nigeria 17% [28]. It is therefore possible that applying CLE would improve GIM detection in these regions as well.

Observation of fluorescein leakage during CLE allows for a simple and objective assessment of mucosal barrier integrity, but its full significance is yet to be determined. It was interesting to note that fluorescein leakage was not associated with non-metaplastic inflamed mucosa. It showed 89% specificity for GIM detection, a novel finding that could be employed as a less invasive optical biopsy technique. Further studies are needed to corroborate these findings.

This study was conducted on asymptomatic HIV positive individuals to avoid bias that might arise from selection based on clinical criteria. Participants in the treated group had been on ART long enough to allow for changes in the gastric milieu that could have been influenced by the use of ART or complete viral suppression. Most of the participants had normal gastric mucosa on WLE, and the occurrence of mucosal abnormalities was similar in the two groups. The proportion of asymptomatic gastric ulcers found in this study was similar to that reported among predominantly HIV negative populations in Iran [29], Korea [30], Taiwan [31] and Zambia [32]. For comparison, we included HIV negative participants as well.

Histologically, NAG was the most common finding, with less than 3% of all the participants having normal histology. The HIV negative group had a significantly higher proportion of individuals with active NAG, although *H*. *pylori* infection was highest in the ART-treated group. This could be an indication of deficient gastric inflammatory response to *H*. *pylori*, as presence of the bacteria did not induce the expected acute inflammation. Other investigators have reported the prevalence of *H*. *pylori* infection to be lower in HIV infection with suggestions of recrudescence of infection in those on ART [33]. Nkuize *et al* reported finding that the histological diagnosis of *H*. *pylori* infection was higher in HIV patients on ART than in those who are ART-naïve [34] and later found that *H*. *pylori* infected HIV patients had lower viral loads and higher CD4 counts [35]. Investigators from Brazil, however, found no association between *H*. *pylori* infection and ART use, HIV viral load or CD4 count [15]. Our study shows a significantly increased burden of *H*. *pylori* infection in the ART treated group. One of the postulated reasons for reduced prevalence of *H*. *pylori* in HIV infection is hypochlorhydria, as high gastric pH reduces its growth. We found a high prevalence of hypochlorhydria in all three groups, although the proportion was lower in the HIV negative group. As the HIV negative participants were drawn from the endoscopy unit, many of them had been prescribed proton pump inhibitors (PPIs) in the days prior to enrolment. Use of PPIs could have increased the occurrence of hypochlorhydria in this group.

Potential limitations of this study include the limited sample size and hospital-based sampling of participants. Further research with mapping of the entire stomach for pre-malignancy using CLE would broaden our understanding of gastric pathology.

## Conclusion

In conclusion, we have shown that CLE is useful for the detection of GIM, and systematic mapping of the antrum underscores the patchy nature of GIM. It may be more sensitive than standard histology for finding GIM. Luminal fluorescein leakage may represent a novel CLE marker for GIM. GIM is common in Zambian adults, and it may be unaffected by ART treatment.

## Supporting information

S1 Dataset This is anonymised data set used for this manuscript: Included are data for HIV positive ART naïve and treated groups and combined HIV positive and HIV negative groups. (XLSX)

S1 Table Comparison of our mapping and the systemic alphanumeric-coded endoscopy. (DOCX)

S2 Table Analysis of the intra-observer variability for the confocal laser endomicroscopy diagnosis of gastric intestinal metaplasia done four weeks apart. (DOCX)

S3 Table Probable factors influencing the number of sites found to have GIM in HIV positive individuals (analysed using non-parametric trend test). (DOCX)

S4 Table Comparison of histology and CLE GIM detection by specific antral area in HIV positive individuals. (DOCX)
