# Supplementary material for: High prevalence of gastric intestinal metaplasia detected by confocal laser endomicroscopy in Zambian adults
Source: PLoS One. 2017 Sep 8;12(9):e0184272. doi: 10.1371/journal.pone.0184272 (PMC5590914; doi:10.1371/journal.pone.0184272)
Supplement: S1 Table — (DOCX) [file pone.0184272.s002.docx]

# Supplementary Table 1: Comparison of our mapping and the Systemic alphanumeric-coded endoscopy

|  | Current study in Zambia | Systemic alphanumeric-coded endoscopy ^22^ |
| --- | --- | --- |
| Lesser curvature | Area 5 | A 8 |
| Anterior wall | Areas 6 and 9 | A 7 |
| Posterior wall | Areas 4 and 7 | A 9 |
| Greater curvature | Areas 8 and 10 | A 10 |
| Incisura | Areas 1, 2 and 3 | Lc 26 |
| Lesser curve, lower third | Area 11 | Lc 25 |
