# Supplementary material for: High prevalence of gastric intestinal metaplasia detected by confocal laser endomicroscopy in Zambian adults
Source: PLoS One. 2017 Sep 8;12(9):e0184272. doi: 10.1371/journal.pone.0184272 (PMC5590914; doi:10.1371/journal.pone.0184272)
Supplement: S2 Table — (DOCX) [file pone.0184272.s003.docx]

Supplementary Table 2: Analysis of the intra-observer variability for the confocal laser endomicroscopy diagnosis of gastric intestinal metaplasia done four weeks apart

|  | **Kappa** | **% Agreement** | **Standard error** | ***P*** |
| --- | --- | --- | --- | --- |
| **Area 1** | 0.82 | 96.9 | 0.12 | <0.000 |
| **Area 2** | 0.86 | 96.8 | 0.13 | <0.000 |
| **Area 3** | 0.69 | 91.9 | 0.12 | <0.000 |
| **Area 4** | 0.63 | 92.1 | 0.12 | <0.000 |
| **Area 5** | 0.78 | 95.5 | 0.12 | <0.000 |
| **Area 6** | 0.85 | 93.7 | 0.12 | <0.000 |
| **Area 7** | 0.69 | 92.2 | 0.12 | <0.000 |
| **Area 8** | 0.77 | 95.5 | 0.12 | <0.000 |
| **Area 9** | 0.70 | 92.4 | 0.12 | <0.000 |
| **Area 10** | 0.49 | 86.9 | 0.12 | <0.000 |
| **Area 11** | 0.84 | 96.6 | 0.13 | <0.000 |
