# Supplementary material for: High prevalence of gastric intestinal metaplasia detected by confocal laser endomicroscopy in Zambian adults
Source: PLoS One. 2017 Sep 8;12(9):e0184272. doi: 10.1371/journal.pone.0184272 (PMC5590914; doi:10.1371/journal.pone.0184272)
Supplement: S3 Table — (DOCX) [file pone.0184272.s004.docx]

Supplementary Table 3: Probable factors influencing the number of sites found to have GIM in HIV positive individuals (analysed using non-parametric trend test)

|  | *P* |
| --- | --- |
| Age | 0.686 |
| Sex | 0.491 |
| ART use | 0.721 |
| Gastric pH | 0.148 |
| Antral atrophy | 0.857 |
| *H.pylori* infection | 0.114 |
| CD4 count | 0.395 |
| Viral load | 0.569 |
