# Supplementary material for: High prevalence of gastric intestinal metaplasia detected by confocal laser endomicroscopy in Zambian adults
Source: PLoS One. 2017 Sep 8;12(9):e0184272. doi: 10.1371/journal.pone.0184272 (PMC5590914; doi:10.1371/journal.pone.0184272)
Supplement: S4 Table — (DOCX) [file pone.0184272.s005.docx]

Supplementary Table 4: Comparison of histology and CLE GIM detection by specific antral area in HIV positive individuals

| **Antral area** | **Intestinal metaplasia on histology** | **Intestinal metaplasia on eCLE** | **OR; 95% CI** | **P** |
| --- | --- | --- | --- | --- |
| Area 1 | 2/54 (4%) | 6/54 (11%) | 7.7; .08-614 | 0.245 |
| Area 2 | 1/56 (2%) | 8/56 (14%) | - | 0.143 |
| Area 3 | 1/55 (2%) | 10/55 (18%) | - | 1.000 |
| Area 5 | 3/59 (5%) | 10/59 (17%) | 12; .05-722 | 0.072 |
| All areas combined | 6/64 (9%) | 21/64 (33%) | 13.1; 1.3-634 | 0.012 |
